# Recent insights into mechanisms of cellular toxicity and cell recognition associated with the ABC family of pore-forming toxins

**DOI:** 10.1042/BST20221409

**Published:** 2023-05-18

**Authors:** Nadezhda A. Aleksandrova, Solace G. Roche, Yu Shang Low, Michael J. Landsberg

**Affiliations:** 1 School of Chemistry and Molecular Biosciences, The University of Queensland, Brisbane, Queensland 4072, Australia; 2 Australian Infectious Diseases Research Centre, The University of Queensland, Brisbane, Queensland 4072, Australia

**Keywords:** ABC toxins, glycans, molecular recognition, pore-forming toxins, receptors

## Abstract

ABC toxins are pore-forming toxins characterised by the presence of three distinct components assembled into a hetero-oligomeric toxin complex ranging in size from 1.5–2.5 MDa. Most ABC toxins studied to date appear to be insecticidal toxins, although genes predicted to encode for homologous assemblies have also been found in human pathogens. In insects, they are delivered to the midgut either directly via the gastrointestinal tract, or via a nematode symbiont, where they attack the epithelial cells and rapidly trigger widespread cell death. At the molecular level, the homopentameric A subunit is responsible for binding to lipid bilayer membranes and introducing a protein translocation pore, through which a cytotoxic effector — encoded at the C-terminus of the C subunit — is delivered. The B subunit forms a protective cocoon that encapsulates the cytotoxic effector, part of which is contributed by the N-terminus of the C subunit. The latter also includes a protease motif that cleaves the cytotoxic effector, releasing it into the pore lumen. Here, we discuss and review recent studies that begin to explain how ABC toxins selectively target specific cells, establishing host tropism, and how different cytotoxic effectors trigger cell death. These findings allow for a more complete understanding of how ABC toxins function in an *in vivo* context, which in turn provides a stronger foundation for understanding how they cause disease in invertebrate (and potentially also vertebrate) hosts, and how they might be re-engineered for therapeutic or biotechnological purposes.

## Introduction

Throughout millions of years of evolution, pathogenic microorganisms have developed a broad array of molecular armaments that help ensure their survival against competing organisms, and aid in establishing and protecting ecological niches [1]. The term virulence factor is a broad, all-encompassing term used to describe such molecules, and includes toxins that attack prey organisms or cells, as well as molecules that help protect against or evade predator organisms and immune responses [2]. Pore-forming toxins (PFTs) collectively are the largest grouping of toxins found in bacteria and while they at first appear diverse in structure and function, paradigms have emerged that define their mechanism of action in a general sense [3]. First, PFTs recognise and bind to a targeted cellular membrane. At some point following this, often triggered by a conformational change in the toxin itself, a transmembrane pore is introduced into the membrane bilayer [4]. The triggers for membrane pore formation can be many and varied, as can be the consequence of pore formation itself [4]. Opening a large, non-selective pore may lead to the loss of essential metabolites or can compromise the structural integrity of the membrane itself, both of which impact cell viability [3]. In many cases, pore formation allows for the passage of associated toxic effector molecules into the target cell, which in turn may trigger more targeted cytotoxic effects [3].

ABC toxins represent an important family of PFTs, members of which were first identified 25 years ago in the bacterium *Photorhabdus luminescens* [5,6]. Related toxins have since been characterised in a range of insect pathogens, such as *Serratia entomophilla* [7], *Xenorhabdus nematophilla* [8] and *Yersinia entomophaga* [9]; as well as in bacterial pathogens of significance to human health, including *Morganella morganii* [10], *Yersinia pestis* [11,12] and *Yersinia pseudotuberculosis* [11,12]. Over the past decade, several landmark studies have begun to elucidate the structures and molecular mechanisms that define this family of PFTs. Here, we briefly review what is currently known about the molecular mechanisms associated with ABC toxin assembly, pore formation and toxin translocation, following which we highlight recent significant advances that enrich our understanding of how these toxins function *in vivo*, including how they specifically recognise their cellular targets, and how they induce potent cytotoxic effects at their cellular destination.

## Structure, assembly and molecular mechanism of ABC toxins — an overview

ABC toxins are large (1.5–2.5 MDa), hetero-oligomeric toxin complexes (Tc) that are prototypically composed of three components referred to hereafter as TcA, TcB and TcC [13]. All three components are required to assemble a complete toxin complex with full toxicity. The TcA component is responsible for membrane binding and transmembrane pore formation [14], while the TcB–TcC component collectively encodes for a cytotoxic enzyme which is encapsulated within a protective, proteinaceous cage [15]. In this respect, ABC toxins can be considered similar to the well-studied binary toxins, which are characterised by separately encoded ‘active’ and ‘binding’ components [16]. Following this analogy, the TcA component can be considered the ‘binding’ component’ while TcB–TcC can be considered the ‘active’ component.

**Figure 1.**
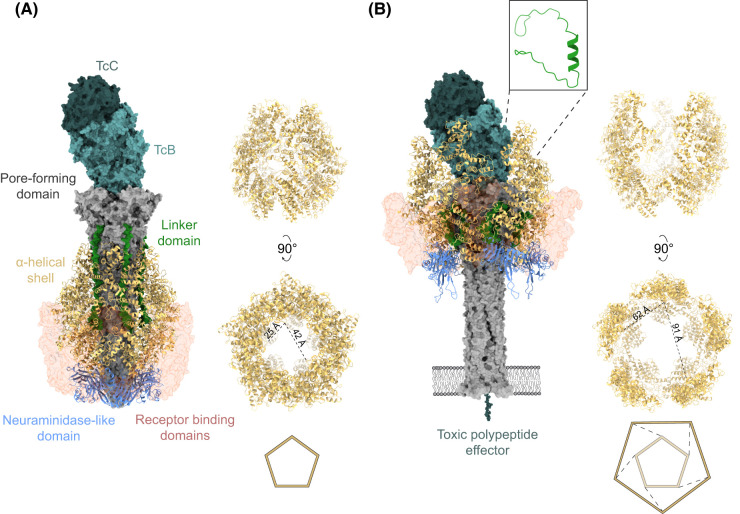
Representative pre-pore (A) and pore form (B) structures of an ABC holotoxin assembly. The figure highlights key structural transitions that occur between the two conformations of the *P. luminescens* PTC3 toxin (PDB ID 6H68 and 6SUF). The TcB–TcC subcomplex (light and dark teal), as well as the α-helical pore (grey), linker (green) and the receptor-binding domains (transparent red) of TcA are shown as surface representations. The α-helical shell (yellow) and neuraminidase-like domains (cornflower blue), which undergo major structural rearrangements, are rendered according to secondary structure. In the pre-pore form, the α-helical shell sheaths the pore-forming domain and the linker adopts a strained conformation with no recognisable secondary structure. In the pore form, the shell has undergone an iris-like expansion, with contacts between the TcA protomers being disrupted. The TcB–TcC subcomplex and the larger top of the pore-forming domain are accommodated within the expanded shell. Folding of the linker into a compact helix drives the pore-forming domain out of its sheath and through an adjacent bilayer membrane.

**Figure 2.**
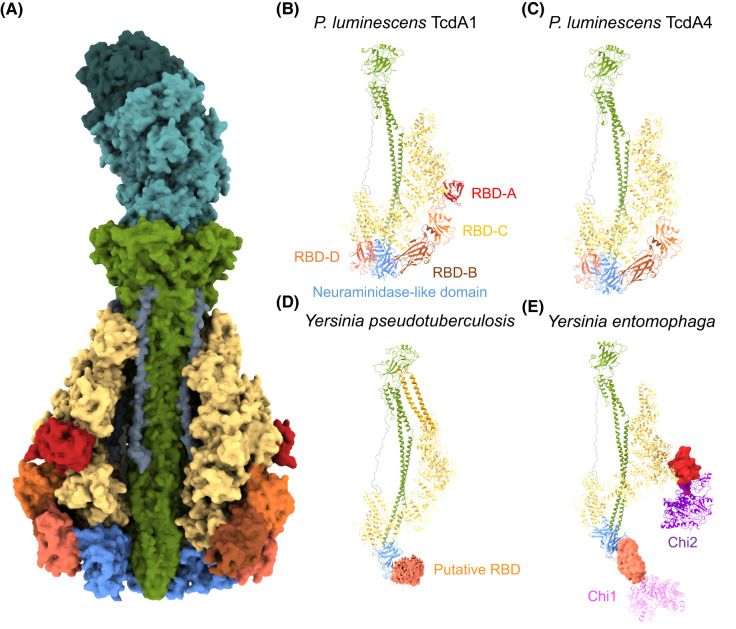
Domain architecture of ABC toxins and diversity of TcA components. (**A**) Cutaway view of the PTC3 holotoxin from *P. luminescens* in the pre-pore state showing the organisation of domains in the toxin. (**B**–**E**) Arrangement of domains within a single TcA protomer from (**B**) *P. luminescens* (TcdA1; PDB ID 6RW6), (**C**) *P. luminescens* (TcdA4; PDB ID 6RWA), (**D**) *Y. pseudotuberculosis* (TcaA–TcaB; PDB ID 6RWB), (**E**) *Y. entomophaga* (YenA1–YenA2–Chi1–Chi2; PDB ID 6OGD). In panel **A**, the TcB and TcC subunits are coloured in light and dark teal, respectively. In all panels, the pore-forming domain is coloured dark green, linker domain is grey, α-helical shell is yellow, and neuraminidase-like domain is blue. In panels **B** and **C**, the Ig-like receptor-binding domains (RBDs) are coloured red, orange, brown and coral for RBD-A, -B, -C and -D, respectively. Note that TcdA4 lacks RBD-A. *Y. pseudotuberculosis* TcaA–TcaB contains a unique, coiled coil domain (panel **D**, dark yellow) and the putative receptor-binding domain (dark orange) is unresolved. The RBD-like domains of YenTc (panel **E**) recruit the additional proteins Chi1 and Chi2 (pink and purple, respectively). The orientation of Chi1 (PDB ID 3OA5) within this structure is ambiguous.

High-resolution structures have been determined for the separate TcA and TcB–TcC components [15,17–19]. More recently, structures of full holotoxin assemblies have provided further insights into how these large complexes function [20,21]. The TcA component is the larger of the two components accounting for ∼80–85% of the complex by mass. It is best described as a three-layered, pentameric assembly, at the centre of which is a long, pre-assembled α-helical pore-forming domain comprised of 10 α-helices (two per protomer) (Figure 1A). These are surrounded by a predominately α-helical shell, which is connected to the pore-forming domain by a long, linker domain with no recognisable secondary structure. The linker extends from the top of the pore-forming domain to an anchor point towards the base of the shell (Figure 1A). The shell is open at the top, allowing the pore-forming domain to attach directly to the TcB–TcC component, and closed at the bottom by a ring of five, conserved, neuraminidase-like domains (one per protomer) (Figure 1A). The bell-shaped structure formed by these two layers is surrounded by a third layer that is far more variable in nature (Figure 2). A varying number of immunoglobulin (Ig)-like receptor-binding domains (RBDs) are inserted into the shell domain (Figure 2) [17]. In some toxins, such as YenTc from *Y. entomophaga*, these domains may not function as bona fide receptor-binding domains on their own, but rather recruit additional protein subunits (endochitinases in the case of YenTc) to the TcA component (Figure 2E) [22,23].

The mode of action associated with ABC toxins is presumed to involve binding of this structure, which represents the soluble, pre-pore configuration, to a targeted cell membrane [21]. Following this, the complex undergoes a dramatic conformational change characterised by an iris-like expansion of the shell and folding of the unstructured linker into a compact α-helix (Figure 1) [21]. The latter drives the pore-forming domain out of the expanded shell and through a nearby membrane bilayer, establishing a transmembrane pore with a diameter of 2–3 nm [21,24].

Unlike the TcA component, the structure of the TcB–TcC component is essentially unchanged between the pre-pore and pore configurations. It binds to the top of the TcA pore in both configurations, and is a β-strand rich, proteinaceous cage that provides a protective environment for stabilising an unfolded toxic effector. (Figure 1) [15,25,26]. The toxic effector is encoded at the C-terminus of TcC, immediately following a *cis*-acting aspartic protease motif, which facilitates the release of the toxic effector from the proteinaceous cage [15]. Upon pore formation, the toxic effector travels through the pore and into the cytoplasm of a targeted cell where it subsequently folds and exerts its toxic effects [20].

## Host cell recognition

Some of the earliest studies of ABC toxins identified that the TcA component was involved in membrane binding and establishing host specificity [8,27,28]. With the determination of the first high-resolution structures, it was proposed that differences in specificity could be attributed to the variable incorporation of Ig-like domains into the TcA shell, identified at the time as putative receptor-binding domains (Figure 2) [19]. The Ig fold is found in many biological contexts, and is most commonly associated with mechanisms of molecular adhesion, including via direct interactions with carbohydrate structures [29,30]. In 2019, Piper et al. [18] provided direct *in vitro* evidence for lectin activity associated with the TcA component of an ABC toxin. They found that the *Y. entomophaga* toxin complex (YenTc) harboured the capacity to bind a variety of glycan structures *in vitro*. Specifically in this case, the lectin activity was localised to the Chi1 and Chi2 subunits (Figure 2E), and glycan microarray experiments indicated that galactose, *N*-acetylgalactosamine, and *N*-acetylglucosamine structures were overrepresented amongst the structures that YenTc interacted with [18].

Soon after, it was concluded that *N*-glycans were necessary for binding and uptake of the *P. luminescens* W14 ABC toxin (PTC3^W14^) [31,32]. In HEK293T cells, it was observed that cellular uptake and toxicity were adversely impacted by PNGase F treatment to remove *N*-linked cell surface glycans, while knockout of *N*-acetylglucosaminyl transferase in HEK293T [32], HeLa [31] and CHO [33] cells had similar consequences. Glycan microarray analyses identified non-insect Lewis X and Y antigens as potential ligands [32,33]. One study conducted a comparative analysis and found no significant affinity for these antigens associated with the insecticidal TcA component XptA1 from *X. nematophila*, nor was there significant affinity seen for the TcA components of ABC toxins from the human pathogens *M. morganii* (Mm-TcdA4) or *Y. pseudotuberculosis* (Yp-TcaA–TcaB), suggesting that the observed interactions may have been specific for PTC3^W14^ [32].

While these studies implicated *N*-glycans in cellular recognition by ABC toxins, if and how the putative RBDs of ABC toxins mediate this remained an open question. Follow-up experiments led Roderer et al. [32] to propose a two-step mechanism for cell surface recognition. This proposal was based on the observation that both a Lewis X-type glycan and heparin/heparan sulphate bound directly to mutually exclusive binding sites within the TcA subunit of PTC3^W14^. Cryo-EM structures revealed that Lewis X binds to RBD-D within the TcA component of PTC3^W14^ [32], an interaction that is mediated by a cluster of aromatic residues that directly interact with the glycan structure. Support for the importance of this interaction to cell intoxication was provided in a separate study, which found that a homologous toxin from the TT01 strain of *P. luminescens* with a divergent RBD-D sequence exhibits a different phenotype to PTC3^W14^. PTC3^TT01^ was still capable of intoxicating *N*-glycan deficient HeLa cells, but when the RBD-D sequence from PTC3^W14^ was grafted onto the equivalent domain of PTC3^TT01^, the loss of function phenotype against *N*-glycan deficient cells was restored [31].

PTC3^W14^ was also noted to interact with sulphated glycosaminoglycans (sGAGs) and unlike the RBD-D mediated interaction with Lewis X, this is proposed to be a less specific interaction. In addition to PTC3^W14^, XptA1, MM-TcdA4 and YptA-YptB are also capable of binding heparin oligosaccharides *in vitro*, and cryo-EM structures of XptA1 and Mm-TcdA4 revealed two different binding surfaces for heparin, neither of which involves a putative RBD [32]. Instead, the oligosaccharide appears to bind directly to the TcA shell, between the RBD-B and neuraminidase domains of XptA1, and in a different location, closer to the expected site of RBD-D, in Mm-TcdA4. The resolution of the ligand in these cryo-EM structures is not sufficient to precisely define the mechanism of recognition, but it is speculated that complementary electrostatic surfaces are primarily responsible. Similarly, genome-wide CRISPR–Cas9 screens identified that multiple genes involved in sGAG biosynthesis were critical determinants of the susceptibility of HeLa cells to intoxication with a hybrid *P. luminescens* ABC toxin that combines a TcA component from a different toxin locus with the TcB and TcC components of PTC3^TT01^ [31]. Either pre-treatment of HeLa cells with surfen (a small molecule that neutralises negative charges on the cell surface), or pre-incubation of the hybrid toxin with several sGAGs (but not unsulfated GAGs) also led to reduced HeLa cell intoxication [31].

Evidence for an insect-specific receptor has very recently emerged with whole genome CRISPR screening identifying the glycoprotein Visgun (Vsg) as an important determinant of the susceptibility of *D. melanogaster* (S2R+) cells to PTC3 intoxication [34]. Vsg knockout rendered these cells less sensitive to PTC3 intoxication. Treatment with *O*-glycosidases (but not with PNGase F, which removes *N*-glycans) reduced the capacity of Vsg to bind the TcA component of PTC3 *in vitro*. Stably transfecting U2OS cells (a bone osteosarcoma cell line, otherwise insensitive to PTC3 at the concentrations used) with *D. melanogaster* Vsg conferred sensitivity to the intoxicating effects of PTC3. Interestingly, U2OS cells transfected with Vsg homologues from mosquitoes and beetles were also sensitive to PTC3, but cells transfected with homologues from moths or humans were not. This study additionally found no evidence for Vsg-dependent toxicity or binding when comparable experiments were conducted with other ABC toxins, including those derived from *M. morganii*, *Y. entomophaga* and *X. nematophila*, leading the authors to conclude that these toxins are likely to have unique physiological targets [34].

How the identification of multiple glycan or glycan-linked receptors for PTC3 in particular relate to one another is somewhat unclear. The identification of *N*-glycan motifs (specifically, non-insect Lewis antigens) and sGAGs as ligands for ABC toxins does offer an explanation for the widely documented observation that, regardless of their native hosts, these toxins often trigger cell death in cultured mammalian epithelial cells. It is possible however that neither of these represent physiologically relevant receptors *in vivo*. The specificity of PTC3 for specific Vsg homologues appears more consistent with this glycoprotein playing a role as a tropism defining-receptor *in vivo*. It is worth mentioning however, that a specific glycan structure responsible for mediating the PTC3–Vsg interaction was unable to be identified [34]. As such, it is possible that similar chemical motifs with slightly differing affinities may play a role in the range of documented interactions. Additionally, it is possible that ABC toxins have built in redundancy to recognise a variety of cell surface epitopes.

## Mechanisms of cytotoxicity

Insecticidal ABC toxins characteristically trigger widespread, apoptotic cell death within the gut epithelia of susceptible hosts [35–38]. While it remains unproven whether ABC toxins are capable of inducing similar disease states in vertebrate hosts, experiments utilising cultured mammalian epithelial cells have demonstrated cytotoxic effects consistent with the pathology observed in insects [32,39]. Examples documented in the current literature indicate aberrant cytoskeleton assembly is a common trigger for cell death, but ABC toxins in general have the potential to function via a variety of molecular mechanisms, a consequence of the fact that the C-terminal domain (CTD) of the TcC component (i.e. the main progenitor of toxicity) is hypervariable. A genome-wide analysis of ABC toxins identified 171 different TcC hypervariable region (HVR) clusters within a dataset derived from an analysis of more than 130 000 genomes. Many of these toxin clusters are homologues of known toxin motifs, including ADP ribosyl transferases (ARTs), amidases, deaminases, deamidases, phospholipases, protein tyrosine phosphatases and peptidases [40]. Direct structural evidence confirming these classifications — and thus a complete understanding of the precise molecular mechanisms responsible for the cellular effects of ABC toxins, has proven unsurprisingly elusive. Available evidence suggests these are unfolded within the structure of the ABC holotoxin assembly [24], and since they are highly toxic enzymes, overexpression of individual toxin domains is challenging. This, in turn, has hindered the full understanding of the molecular mechanisms that lead to cell toxicity.

**Figure 3.**
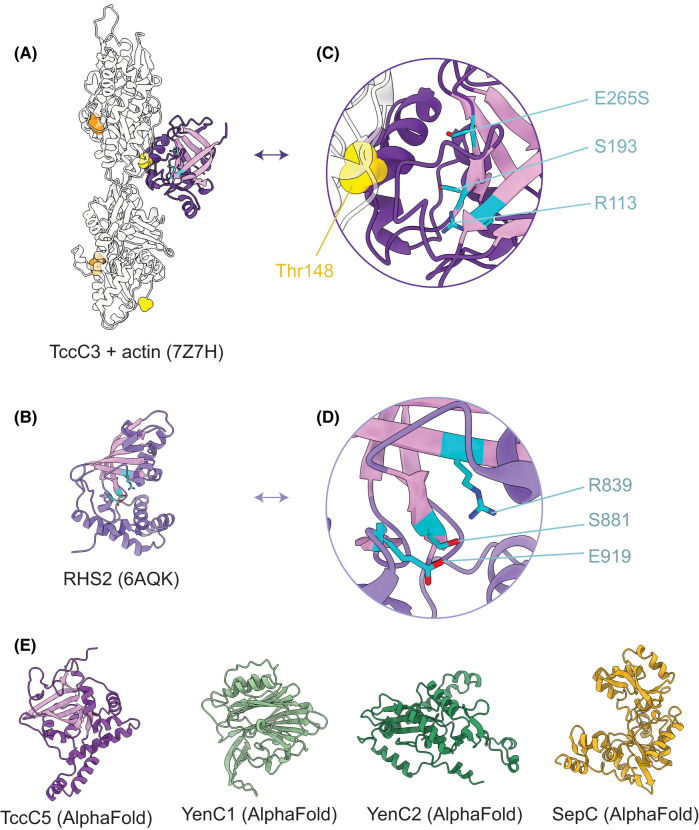
Examples of cytotoxic effectors associated with ABC toxins. (**A**) Structure of the TccC3^CTD^ in complex with two actin subunits (PDB ID 7Z7H). The structure is extracted from a longer actin filament, which is the molecular target of TccC3. Actin residues threonine-148 (yellow) — the residue modified by TccC3 — and arginine-177 (orange) — the residue modified by canonical R-S-E motif-containing ARTs — are highlighted as spheres. The conserved β-sheets of the ART fold are coloured in pink and other structural elements are coloured purple. (**B**) Structure of RHS2 (PDB ID 6AQK). (**C** and **D**) Zoomed in view showing the sidechains (highlighted as blue sticks) that comprise the R-S-E motif for TccC3 and RHS2, respectively. In the TccC3 structure, the catalytic E265 has been mutated to serine in order to inhibit enzymatic activity and trap the protein in the actin-bound form. (**E**) Predicted structures of TcC HVRs of selected ABC toxin components highlight the diversity of folds encoded for by different toxins, but also infer similarities to established classes of cytotoxic effectors associated with other bacterial toxin systems. All structures were predicted using the AlphaFold implementation deployed on the Australian BioCommons Galaxy Australia platform.

To date, the only structurally characterised examples of toxic effectors associated with ABC toxins are ARTs, a widely distributed class of enzymes among bacteria that frequently contribute to the virulence of pathogenic species [41]. The structure of the HVR of one of the two TcC components that are alternately associated with the *P. luminescens* PTC3 toxin (TccC3^CTD^) was recently solved by NMR spectroscopy [25]. Similar to other ARTs, TccC3^CTD^ has a conserved NAD^+^-binding region, as well as core structural features that define the ART fold; a region of β-sheets housing a characteristic R-S-E motif and stabilised by a conserved pair of hydrogen bonds. However, the overall ART fold of TccC3^CTD^ is considered non-canonical due to the unconserved nature of its α-helical region. The importance of this non-canonical structural motif was subsequently shown to play a critical role in binding to F-actin (Figure 3A,B), which is an unusual feature of TccC3^CTD^. Other well-characterised R-S-E motif ARTs ADP-ribosylate actin monomers, primarily by modifying actin at arginine-177. This in turn alters actin turnover by either promoting or inhibiting filament formation [42]. In contrast, TccC3^CTD^ does not ADP-ribosylate monomeric actin, but instead binds to the interface between two actin subunits within a filament and transfers an ADP-ribose to threonine-148 [25,39]. Modifying this region obstructs binding of actin depolymerising factors such as cofilin, resulting in more stable filaments and aberrant actin turnover.

Not all ARTs disrupt the cytoskeleton by directly targeting actin. For example, through functional studies it has been shown that TccC5^CTD^, another toxic effector of *P. luminescens* with a predicted ART fold, ADP-ribosylates Rho GTPases [39]. This modification constitutively activates the GTPase leading to aberrant stress fibre formation and cytoskeletal dynamics. TccC5^CTD^ is predicted to have a structure similar to other bacterial toxins targeting Rho GTPases such as the Vis toxin from *Vibrio splendidus* (Figure 3E).

Like the *P. luminescens* PTC3 toxin locus, *Y. entomophaga* also contains two, alternately encoded TcC components within its YenTc toxin locus. The HVR of YenC1 (YenC1^CTD^; Figure 3E) is predicted to share most structural similarity with the family of Rho-activating cytotoxic necrotising factor (CNF) proteins from *Y. pseudotuberculosis* and *E. coli*. Interestingly, while CNFs are not ARTs, they typically target Rho GTPases and thus YenC1 and TccC5 may have a common cellular target. The predicted structure of YenC2^CTD^ on the other hand most closely resembles that of eukaryotic zinc-dependent cytidine deaminases like the ones from *Aspergillus terreus* and *Mus musculus*. While bacterial cytidine deaminases do represent an established class of toxic effectors, it is worth acknowledging speculation that TcC HVRs may not exclusively be toxic effectors, and that some may play additional important roles in the life cycle of the bacterium [40].

Curiously, the genome of *Y. entomophaga* includes a third gene that encodes a TcC-like protein named RHS2, but this gene is located outside the YenTc toxin locus and is not in close proximity to any other predicted ABC toxin components. The structure of RHS2^CTD^ has been solved by X-ray crystallography (PDB ID: 6AQK), and like TccC3^CTD^, it features an ART fold (Figure 3C,D). However, the RHS2^CTD^ does not share the unique arrangement of α-helices that is a feature of TccC3^CTD^, and instead more closely resembles the canonical R-S-E motif-containing ARTs, including the SpvB protein from *Salmonella enterica.* Therefore, it seems most likely that the cellular target of RHS2 is monomeric actin. ‘Orphan’ toxic effectors are frequently found in association with other multicomponent toxin systems [43–45], and it is possible that RHS2 functions as an ‘orphan’ TcC protein, capable of recombining with the other components of YenTc.

Owing to the fact that a remarkably small number of HVRs have been structurally characterised, these examples underrepresent the diversity of virulence mechanisms that are potentially associated with ABC toxins. There is emerging evidence that this diversity may additionally impact the way ABC toxins function, for example by playing roles in defining host specificity. The SepABC toxin locus from *Serratia entomophila* contains only a single TcC component (SepC), and SepC^CTD^ is predicted to be an adenylate cyclase (Figure 3E) with similarity to the anthrax toxin edema factor. The susceptible host profile of this toxin is relatively narrow [37], but a recent study into the virulence of different *Serratia* subspecies found that in some species, inclusion of more than one TcC protein led to these gaining additional susceptible insect hosts [28]. Originally assumed to be a mechanism via which resistance is overcome in established hosts, it therefore appears that diversifying the range of TcC components within a single organism (e.g. through gene duplication) may therefore represent an important mechanism to which host tropism is also linked.

## Open questions and future directions

Collectively, the studies highlighted here complement earlier findings that have defined the assembly and mechanism of ABC toxins, extending these into a physiological context where the determinants of cellular tropism and host cell recognition have begun to be elucidated, alongside detailed descriptions of the cellular mechanisms of toxicity. Despite these advances, further questions still remain to be answered. Further studies are necessary to identify and characterise more toxins and their respective cellular receptors. These in turn will help to identify the extent to which mechanisms of host recognition are (or are not) conserved. This will improve our understanding of how ABC toxin-encoding bacteria establish host tropism, and in turn inform a clearer picture of one mechanism via which bacteria gain advantages over competing organisms within ecological niches.

The TcC effectors characterised in recent studies represent a small fraction of a diverse group of proteins that could differ greatly in terms of their molecular structure, as well as the mechanisms via which they initiate cell death. Considering the large numbers of toxic effectors that are present in bacteria, many remain uncharacterised in terms of their structure and function. Understanding the cytotoxicity mechanisms associated with novel effectors will provide a more complete understanding of their cellular function, and may also be beneficial in the selective engineering of these proteins for biotechnological applications [26].

Finally, there are still aspects of the mechanism via which ABC toxins are activated *in vivo* that remain to be fully elucidated. It is currently accepted that the α-helical shell is stabilised in the pre-pore form by a largely undefined network of electrostatic interactions that — for most, but not all ABC toxins [17,18] — is disrupted *in vitro* at either low (<5) or high (>11) pH environments, triggering conversion to the pore form. *In vivo*, the former might be encountered within an acidifying endosome [23], while the latter would potentially be encountered in alkaline micro-environments at the cell surface, particularly in the insect midgut where many of these toxins are known to be active [46]. It is plausible that any destabilising effect could be enhanced by receptor binding *in vivo*, through allosteric effects for example. Curiously, there is also evidence suggesting that specific proteases might play an important role in activating the *P. luminescens* PTC3 toxin, which exhibited enhanced cell binding following treatment with either PrtA1 or collagenase and reduced cell intoxication in the presence of cathepsin inhibitors [47]. Thus, the mechanism of toxin activation *in vivo* may be more complex than what is suggested from *in vitro* studies alone and warrants further investigation.

## Perspectives

- ABC toxins are a recently identified family of pore-forming virulence factors found in pathogens of insects and animals. Their mode of action represents an understudied mechanism of bacterial pathogenesis.
- ABC toxins package and deliver a variety of toxic effectors, giving rise to a diverse range of cellular mechanisms for triggering cell death. Glycans are increasingly implicated in the binding of ABC toxins to their cellular targets in a way that defines host tropism. Therefore, ABC toxins possess the capability to execute a highly specific, potent and targeted cytotoxic effect.
- Further investigations should focus on broadening our understanding of the molecular diversity that exists within the ABC toxin family, as well as exploring their exciting potential to be deployed or re-engineered for use as novel biopesticides, or in other bespoke biotechnological applications. The current literature provides a limited view of the diversity of mechanisms that are potentially involved in host cell recognition, toxin activation and cytotoxicity and a more complete and thorough investigation of these phenomena is essential.

## Open Access

Open access for this article was enabled by the participation of University of Queensland in an all-inclusive *Read & Publish* agreement with Portland Press and the Biochemical Society under a transformative agreement with CAUL.

## Abbreviations

ADP: adenosine diphosphate
ART: ADP ribosyl transferases
CTD: C-terminal domain
HVRs: hypervariable regions
Mm-TcdA4: *Morganella morganii* TcdA4
NAD+: nicotinamide adenine dinucleotide
NMR: nuclear magnetic resonance
PFTs: pore-forming toxins
PTC3^W14^: *Photorhabdus luminescens* W14 Tc toxin
RBDs: receptor-binding domains
sGAGs: sulphated glycosaminoglycans
Tc: toxin complexes
Xn-XptA1: *Xenorhabdus nematophila* XptA1
YenTc: *Yersinia entomophaga* toxin complex
Yp-TcaA–TcaB: *Yersinia pseudotuberculosis* TcaA–TcaB
